# Fabrication and Photocatalytic Properties of Zinc Tin Oxide Nanowires Decorated with Silver Nanoparticles

**DOI:** 10.3390/nano12071201

**Published:** 2022-04-03

**Authors:** Jia-Chi Su, Tsung-Lin Hsieh, Shu-Meng Yang, Shao-Chun Chao, Kuo-Chang Lu

**Affiliations:** 1 Department of Materials Science and Engineering, National Cheng Kung University, Tainan 701, Taiwan; n56081412@ncku.edu.tw (J.-C.S.); n56091255@gs.ncku.edu.tw (T.-L.H.); n56074287@gs.ncku.edu.tw (S.-M.Y.); n56104375@gs.ncku.edu.tw (S.-C.C.); 2 Core Facility Center, National Cheng Kung University, Tainan 701, Taiwan

**Keywords:** zinc tin oxide, nanowires, chemical vapor deposition, surface modification, photocatalysis, reactive radical species

## Abstract

With the continuous advancement of high-tech industries, how to properly handle pollutants has become urgent. Photocatalysis is a solution that may effectively degrade pollutants into harmless molecules. In this study, we synthesized single crystalline Zn_2_SnO_4_ (ZTO) nanowires through chemical vapor deposition and selective etching. The chemical bath redox method was used to modify the ZTO nanowires with Ag nanoparticles to explore the photocatalytic properties of the nanoheterostructures. The combination of the materials here is rare. Optical measurements by photoluminescence (PL) and UV–Vis show that the PL spectrum of ZTO nanowires was mainly in the visible light region and attributed to oxygen vacancies. The luminescence intensity of the nanowires was significantly reduced after modification, demonstrating that the heterojunction could effectively reduce the electron-hole pair recombination. The reduction increased with the increase in Ag decoration. The conversion from the UV–Vis absorption spectrum to the Tauc Plot shows that the band gap of the nanowire was 4.05 eV. With 10 ppm methylene blue (MB) as the degradation solution, ZTO nanowires exhibit excellent photodegradation efficiency. Reusability and stability in photodegradation of the nanowires were demonstrated. Photocatalytic efficiency increases with the number of Ag nanoparticles. The main reaction mechanism was confirmed by photocatalytic inhibitors. This study enriches our understanding of ZTO-based nanostructures and facilitates their applications in water splitting, sewage treatment and air purification.

## 1. Introduction

Previous studies indicate that industrial dyes and textile dyes are the largest organic pollutants, causing increasing environmental pollution [1,2]. About 10–20% of the world’s dye production is lost during the dying process and discharged into textile wastewater. Due to water pollution, advanced oxidation processes (AOPs) have been rapidly developed as an innovative water treatment technology [3,4]. The principle of AOPs is to generate highly reactive transition substances based on the catalytic properties of materials, such as H_2_O_2_, ·OH^−^ and ·O_2_^−^ for organic compounds which are difficult to decompose. Semiconductors are often used as catalysts in AOPs, such as TiO_2_, ZnO and Fe_2_O_3_, which have been demonstrated to degrade organic compounds effectively, finally mineralizing them into harmless carbon dioxide and water [5].

Zinc tin oxide, also known as ZTO, is a non-toxic ternary oxide semiconductor (A_2_BO_4_, inverse spinel structure) with high conductivity, low visible light adsorption, high electron mobility and excellent thermal stability. Ternary metals have higher chemical stability as compared to binary metal oxides. ZTO nanostructures have been fabricated for various important applications, including optical sensors [6,7], gas sensors [8,9], dye-sensitized solar cells [10,11] and photocatalysts [12,13,14]. In the past few years, different techniques have been applied in synthesizing one-dimensional ZTO nanostructures, including the hydrothermal method [15,16], CVD [7,17] and the template method [18], among which CVD is the best choice since it is more economical and environmentally friendly [19,20].

In terms of photocatalytic applications, surface modification of precious metals is frequently used to increase photocatalytic efficiency, such as Ag [21,22,23], Pd [24] and Pt [25], which can form Schottky contact through the junction between the metal and semiconductor and lower the recombination efficiency of electron-hole pairs to enhance photocatalytic properties.

In this work, we studied ZTO NWs and Ag-ZTO NWs as efficient photocatalysts in water treatment for environmental pollution. We synthesized ZTO NWs by the chemical vapor deposition (CVD) method via the vapor–liquid–solid (VLS) route and modified the nanowires using the chemical bath redox method. The 1 at% Ag-ZTO NWs and 3 at% Ag-ZTO NWs were fabricated with different amounts of AgNO_3_. All three kinds of nanowires were characterized by SEM, TEM, XRD, EDS, XPS and PL to identify their structures and phases.

## 2. Materials and Methods

### 2.1. Synthesis of ZTO and Ag-ZTO Nanowires

ZTO NWs were synthesized with a three-zone tube furnace via catalyst-assisted chemical vapor deposition (CVD) through a vapor–liquid–solid (VLS) growth mechanism. The mixture of zinc oxide powder (0.12 g) (Alfa Aesar, Ward Hill, MA, USA purity 99.9%) and active charcoal (0.12 g) (Sigma-Aldrich, St. Louis, MO, USA) was loaded into an alumina boat and placed in the second heating zone. Tin oxide powder (0.03 g) (Alfa Aesar, Ward Hill, MA, USA purity 99.9%) and active charcoal (0.03 g) were mixed with the same ratio and placed in the third heating zone. A 10 nm-thick Au layer on the silicon substrate was deposited by the E-beam evaporation system. Nanowire growth was based on the vapor−liquid−solid (VLS) mechanism with Au as catalyst. The alumina boat of sonically cleaned Si (100) substrates with a 10 nm thin Au layer was placed downstream outside the third heating zone. The temperatures of the second and third heating zones were raised to 955 and 905 °C for 1.5 h, respectively. Argon gas was introduced with the flow rate of 100 sccm. After the reaction, the furnace was cooled down to room temperature naturally. The single phase ZTO NW substrate was immersed in 12 mM sodium borohydride (NaBH_4_) (98+%, Acros Organics, Morris Plains, NJ, USA) solution, which was then titrated with silver nitrate solution (0.01 and 0.03 g) of deionized water (pH = 7, ionic strength = 0.001 M) and AgNO_3_ to form Ag-ZTO NWs as shown in Figure S1. 

### 2.2. Electrical Measurements

To fabricate the nanodevice for single nanowire electrical resistivity measurements, ZTO NWs and Ag-ZTO NW substrates were immersed into deionized water (pH = 7, I = 0.001 M) and sonicated to separate the nanowires from the substrate. The solution with nanowires was dripped on an as-prepared Si/SiO_2_ substrate with four independent Ag electrodes with 400 nm-thick platinum to which the nanowire was connected by a focus ion beam (FIB, FEI Nova-200 NanoLab Compatible, Hillsboro, OR, USA). The electrical m easurements of a single nanowire here were based on the method previously reported by Gu et al. [26], which can eliminate the voltage drop and contact resistance during measurement to obtain more accurate results. The schematic illustration and setup of the electrical measurements are shown in Figure S5. 

### 2.3. Photodegradation

The photodegradation performance of ZTO and Ag-ZTO NWs was evaluated with 10 ppm methylene blue (0.5 g/100 mL) (Sigma-Aldrich, St. Louis, MO, USA) in deionized water (pH = 7, I = 0.001 M) as shown in the experimental setup of Figure 5g. The sample was placed at the bottom of a beaker perpendicular to the simulated solar light lamp. We stirred 5 mL MB in the beaker using a magnetic stirrer in the darkroom for 30 min to reach an adsorption–desorption balance for UV–Vis absorption measurement as the MB initial concentration C_0_. During the illumination of the Xe lamp (105 W/m^2^, Oriel^®^ LCS-100^TM^ Small Area Sol1A), we took 0.5 mL MB per 30 min for the UV–Vis absorption measurement as C_t_. The photodegradation efficiency was then calculated according to Equation (1) [27]:

(1) $$\mathrm{Degradation}\left(\%\right)=\left(1-\frac{C_{t}}{C_{0}}\right)\cdot 100\%$$

### 2.4. Radical Trapping Experiment

To understand the major reactive species in the MB photodegradation experiment, radical scavengers were added to inhibit different radical work during the photodegradation process. In this study, 1 mM ethylenediaminetetraacetic acid (EDTA) was used to inhibit h+, 1 mM tert-butanol (TBA) was used to inhibit ·OH and 1 mM 1,4-benzoquinone (BQ) was used to inhibit ·O_2_^−^. Other procedures were the same as the photodegradation experiment. Lower photodegradation efficiency after adding a specific scavenger corresponds to higher importance of the reactive species.

### 2.5. Characterization

Morphologies and structures of ZTO and Ag-ZTO NWs were characterized by field-emission scanning electron microscopy (FESEM, Hitachi SU8000, Tokyo, Japan ), X-ray di ffractometry (XRD, Bruker D8 Discover with GADDS, Fitchburg, WI, USA), transmission electron microscopy (TEM, JEOL-2100F, CS STEM, Tokyo, Japan), Energy-dispersive X-ray spectroscopy (EDS) and X-ray photoelectron spectroscopy (XPS, PHI 5000 VersaProbe, ULVAC-PHI, Kanagawa, Japan). Photoluminescence (PL, HORIBA LabRAM HR, Longjumeau, France) and UV–Vis spectrophotometry (PerkinElmer LAMBDA 950, Waltham, MA, USA) were utilized for optical and photocatalysis properties.

## 3. Results and Discussion

### 3.1. Morphology and Structure Analysis of ZTO NWs and Ag-ZTO NWs

#### 3.1.1. SEM and XRD Analysis

**Figure 1:**
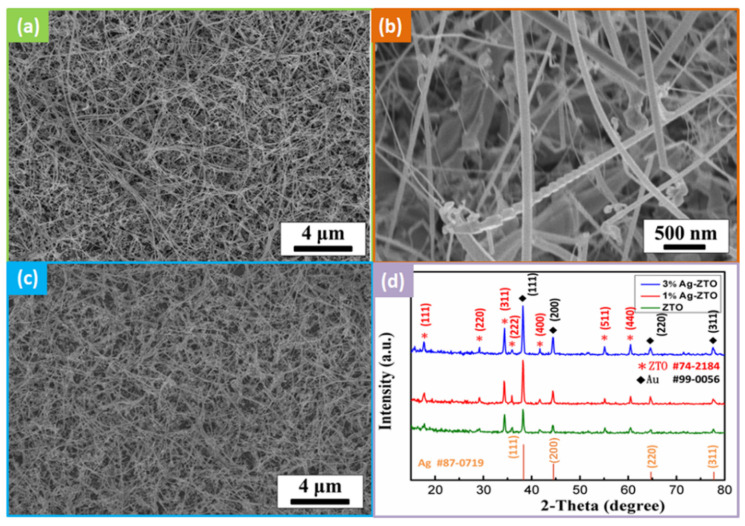
Morphologies of ZTO NWs and Ag-ZTO NWs. (**a**) Low magnification SEM image of ZTO NWs. (**b**) High magnification SEM image of ZTO NWs. (**c**) Low magnification SEM image of Ag-ZTO NWs. (**d**) XRD pattern of ZTO NWs and Ag-ZTO NWs.

It has been difficult to fabricate pure ternary nanowires with a furnace; for example, synthesis of Zn_2_SnO_4_ (ZTO) nanowires frequently comes with simultaneous synthesis of ZnO and SnO_2_ [28]. Therefore, ZnO/ZTO NWs were chosen for synthesis, and we used 12.5 mM aqueous hydrochloric acid (HCl) to remove the ZnO phase. Figure S2a,b show the SEM images of before and after the selective etching process; Figure S1c shows the XRD pattern after the selective etching process, indicating that we successfully obtained single phase ZTO NWs. Figure 1a,b show the high-density ZTO NWs had a length of over 10 μm and diameter of about 40–80 nm. Figure 1c is the SEM image after modification of Ag nanoparticles. Figure 1d shows the XRD patterns for ZTO NWs, 1 at% Ag-ZTO NWs and 3 at% Ag-ZTO NWs; the peaks located at 17.7° (111), 29.2° (220) and 34.4° (311) correspond to ZTO (JCPDS no.74-2184), while the peaks at 38.3° (111) and 44.5° (200) correspond to Au (JCPDS no.99-0056) because of the VLS growing mechanism. Since the diffraction peaks of Ag (JCPDS no. 87-0719) and Au are almost the same, the diffraction peaks of Au appear wider after modification of Ag.

#### 3.1.2. HRTEM and EDS Analysis

**Figure 2:**
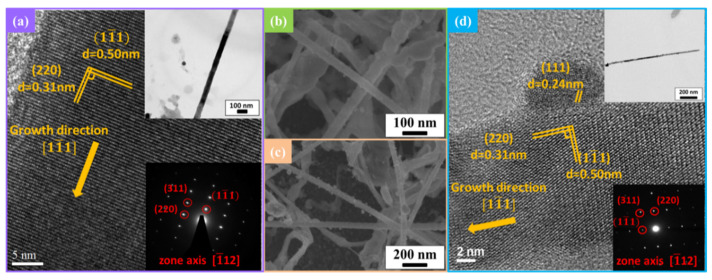
Morphology and structure analysis of ZTO NWs and Ag-ZTO NWs. (**a**) HRTEM image of ZTO NWs. (**b**) SEM image of 1 at% Ag-ZTO NWs. (**c**) SEM image of 3 at% Ag-ZTO NWs. (**d**) HRTEM image of Ag-ZTO NWs.

Figure 2a is a HRTEM image of the ZTO nanowire, where the interplanar spacings, 0.5 and 0.31 nm, correspond to (111) and (220) planes of Zn_2_SnO_4_, and the inset is the selected area electron diffraction (SAED) pattern corresponding to the diffraction peaks in the XRD analysis. Figure 2b,c are SEM images of 1 at% Ag-ZTO NWs and 3 at% Ag-ZTO NWs, respectively; more Ag nanoparticles with a diameter of 5–20 nm were on the surface of the latter. Figure 2d is a HRTEM image of Ag-ZTO NW, where the interplanar spacings of the nanowire are the same as those of the ZTO NW in Figure 2a; additionally, attached on the surface is a particle with an interplanar spacing of 0.24 nm, corresponding to the (111) plane of Ag. Based on the XRD and TEM analysis, the nanoparticles were confirmed to be Ag. Figure S4a,b show the EDS studies of 1 at% Ag-ZTO and 3 at% Ag-ZTO NW; Figure S4c,d show the mapping of 1 at% Ag-ZTO and the 3 at% Ag-ZTO NW. EDS analysis indicates that ZTO NWs are nonstoichiometric compounds, but XRD and XPS studies confirm that the structure and valence of elements are correct.

#### 3.1.3. XPS Analysis

**Figure 3:**
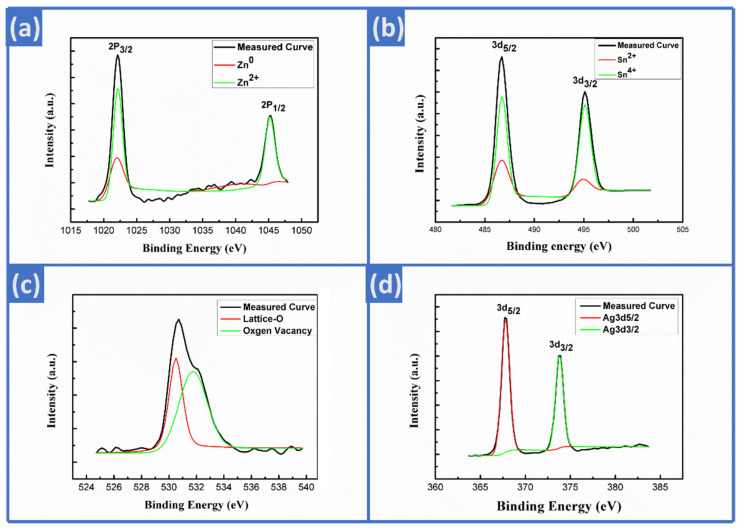
XPS analysis for Ag-ZTO NWs. (**a**) Zn 2p, (**b**) Sn 3d, (**c**) O 1s, (**d**) Ag 3d.

XPS analysis was conducted to further investigate the composition of Ag-ZTO NWs. The valence states of the elements in the nanowires can be confirmed by XPS curve fitting as shown in Figure 3a–d. Figure 3a shows the 2p_1/2_ and 2p_3/2_ orbitals of zinc, the binding energies of which were 1021.7 and 1044.7 eV, coherent with the previously reported energy difference of 23 eV [29]; the two characteristic peaks correspond to two valence states, Zn^0^ and Zn^2+^. Figure 3b reveals two characteristic peaks of 3d_5/2_ and 3d_3/2_ orbitals of tin, which were from Sn^2+^ and Sn^4+^, the binding energies of which are about 486.7 and 495.1 eV, respectively. The binding energy of the 3d orbitals between Sn^2+^ and Sn^4+^ states is very close. It is known from the previous report [30] that the binding energy for Sn^4+^ is bigger than that for Sn^2+^, demonstrating that the blue line denotes Sn^4+^, while the red line denotes Sn^2+^. Figure 3c indicates that the oxygen binding energy of ZTO NWs mainly resulted from oxygen in the lattice and oxygen vacancies, the binding energies of which were 530.8 and 532 eV, respectively [31]. After surface modification, Figure 3d shows the binding energies of 3d_5/2_ and 3d_3/2_ orbitals of silver were 367.6 and 373.6 eV, respectively [32]. 

#### 3.1.4. UV–Vis and PL Analysis

**Figure 4:**
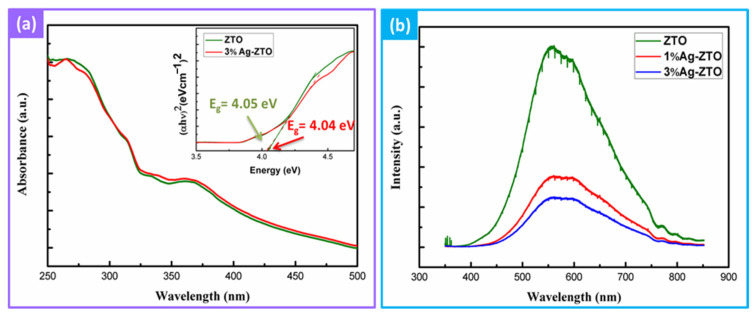
Optical properties of ZTO NWs and Ag-ZTO NWs. (**a**) UV–Vis spectra (**b**) PL spectra.

To explore the optical properties of ZTO NWs and Ag-ZTO NWs, it is important to determine the band gap. Figure 4a is the room temperature UV–Vis absorption spectra of ZTO NWs and 3 at% Ag-ZTO NWs; both had a significant increase in absorption at a wavelength less than 325 nm. The absorption spectrum feature at 350–450 nm corresponds to impurities or oxygen vacancy levels. The band gap of the nanowires was calculated with Tauc’s equation [12]:

(αhν)^1/n^ = A(hν − E_g_) (2)

where α is the adsorption coefficient of the material, A is a proportionality constant and n is a constant exponentially different based on the types of semiconductors, being 1/2 for direct bandgap semiconductors. The inset of the UV–Vis spectra is the Tauc Plot. According to the tangent of the diagram, the band gaps of ZTO NWs and 3 at% Ag-ZTO NWs were 4.05 and 4.04 eV, which are close to the previously reported value (3.92 eV). The wider band gap for the ZTO NWs may be attributed to their smaller sizes [14]. The similar absorption edges before and after Ag modification indicate that their bandgap energy difference is negligible [22,23].

Figure 4b shows the PL spectra for a 325 nm laser for ZTO NWs and Ag-ZTO NWs at room temperature, exhibiting a wide emission band from 425 to 750 nm with a central wavelength of 580 nm. Various emission peaks might contribute to the wide PL spectrum. The green fluorescent signal around the 560 nm peak was emitted from the conduction band (CB) to oxygen vacancies (V^+^_0_) [33]. The orange fluorescent signal around the 590 nm peak was attributed to oxygen vacancies and interstitial tin vacancies. The red fluorescent signal around the 650 nm peak resulted from interstitial zinc and tin vacancies [34]. In the PL analysis, oxygen vacancies mostly resulted from the large surface area of nanowire and lack of oxygen due to nanowire growth by three zone furnaces; thus, many oxygen vacancies existed in the nanowires. During nanowire growth, oxygen vacancies tended to diffuse from the interior to the surface; thus, the oxygen vacancy concentration on the surface was greater than that in the interior of the nanowires [35]. The excited electrons were captured by the metastable energy band caused by the oxygen vacancies and then released to recombine with holes [29]. Therefore, oxygen vacancies improved photocatalytic performance by reducing the recombination rate of electron-hole pairs. After Ag modification, the photoluminescence intensity decreased dramatically, the reason behind which is discussed in the photodegradation section.

### 3.2. Growth Mechanism

Figure S3 is the schematic illustration of the ZTO NW growth mechanism. Zinc oxide powder and tin oxide powder were reduced to Zn_(v)_ and Sn_(v)_ through the carbothermic reduction method based on Equations (3) and (4). At 900 °C, the Au catalyst on the surface of the silicon substrate was transformed to liquid and reacted with Zn_(v)_ and Sn_(v)_ to form an Au–Zn–Sn liquid alloy. As the carrier gas delivered more precursors to the substrate, the Au–Zn–Sn liquid alloy was supersaturated, precipitated and oxidized into ZTO NWs. The ZTO nanowires grew longer with the continuous precipitation of the droplets according to Equation (5):

(3) $${\mathrm{ZnO}}_{\left(s\right)}+C_{\left(s\right)}\overset{\Delta }{\to }{\mathrm{Zn}}_{\left(v\right)}+{\mathrm{CO}}_{\left(v\right)}$$

(4) $${\mathrm{SnO}}_{2\left(s\right)}+C_{\left(s\right)}\overset{\Delta }{\to }{\mathrm{Sn}}_{\left(v\right)}+{\mathrm{CO}}_{2\left(v\right)}$$

(5) $${\mathrm{Au}}_{\left(l\right)}+2{\mathrm{Zn}}_{\left(v\right)}+{\mathrm{Sn}}_{\left(v\right)}+2O_{2\left(v\right)}\overset{\Delta }{\to }{\mathrm{Zn}}_{2}{\mathrm{SnO}}_{4\left(s\right)}+{\mathrm{Au}}_{\left(s\right)}$$

### 3.3. Electrical Measurements of Single ZTO NW and Ag-ZTO NW

Figure S5a shows the schematic illustration of the electrical measurements and Figure S5b–d are SEM images of ZTO NW, 1 at% Ag-ZTO NW and 3 at% Ag-ZTO NW connected to Ag electrodes, respectively. Each single nanowire was measured 8 times, including R13, R14, R23 and R24, by applying positive and negative voltages. The I-V measurements for ZTO NW, 1 at% Ag-ZTO NW and 3 at% Ag-ZTO NW are shown in Figures S6–S8, respectively. Based on Table 1, the resistivity of ZTO NW here was 6.01 × 10^−5^ Ω·m, which is better than previous studies [36]; the resistivity of 1 at% Ag-ZTO NW and 3 at% Ag-ZTO NW was 2.1 × 10^−4^ and 4.3 × 10^−4^ Ω·m, indicating the resistivity increase after surface modification. 

### 3.4. Photocatalytic Properties of ZTO NWs and Ag-ZTO NWs

#### 3.4.1. Photodegradation Activities

**Figure 5:**
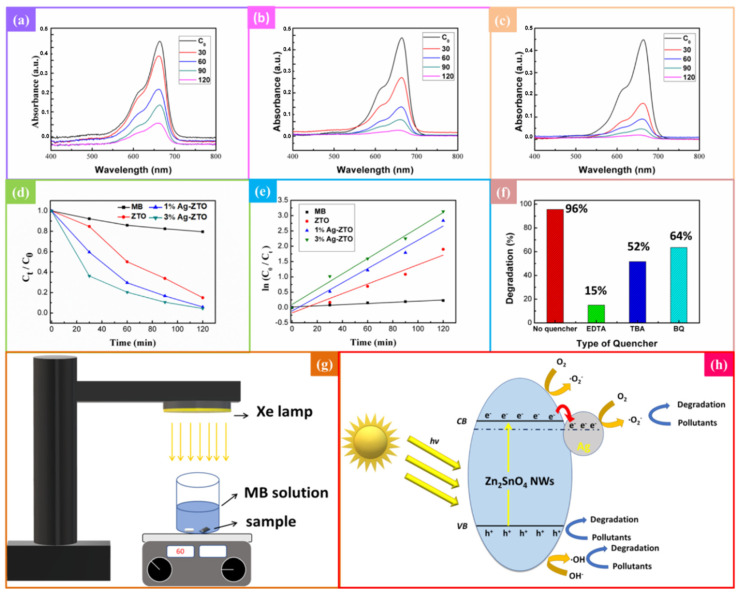
Photocatalytic properties of ZTO NWs and Ag-ZTO NWs were evaluated with 10 ppm methylene blue in deionized water (pH = 7, I = 0.001 M). The UV–Vis absorbance spectra of (**a**) ZTO NWs, (**b**) 1 at% Ag-ZTO NWs, (**c**) 3 at% Ag-ZTO NWs towards MB dye. (**d**,**e**) Comparison of photocatalytic activities between ZTO NWs and Ag-ZTO NWs. (**f**) Comparison of photocatalytic activities among different scavengers for 3 at% Ag-ZTO NWs. (**g**) Schematic illustration of photodegradation experimental setup. (**h**) Schematic illustration for photodegradation mechanism of Ag-ZTO NWs.

Methylene blue (MB) is a heterocyclic dye with wide industrial applications, being frequently used for coloring paper, as a temporary hair colorant and for dyeing cottons, wools and so on. Additionally, it has been extensively used in human and veterinary medicine for several therapeutic and diagnostic procedures. It cannot be degraded through the conventional water treatment process due to its complex aromatic structures, hydrophilic nature and high stability against light, temperature, water, chemicals, etc.; thus, it may cause substantial environmental pollution. Although MB is not considered to be a very toxic dye, it can result in very harmful effects on living things, such as breathing difficulties, vomiting, diarrhea and nausea. Therefore, many efforts have been made to remove MB from water [37,38], and we chose it to investigate the photodegradation activities of ZTO NWs and Ag-ZTO NWs. MB has a very strong characteristic peak at 663 nm [39]; the efficiency of photodegradation can be calculated based on the change of the absorption peak of MB. Figure 5a shows the UV–Vis absorption spectra of MB degraded by ZTO NWs, while Figure 5b,c show the UV–Vis absorption spectra of MB degraded by 1 at% Ag-ZTO NWs and 3 at% Ag-ZTO NWs. It can be seen that the absorption spectra of 3 at% Ag-ZTO NWs have the most obvious change; therefore, 3 at% Ag-ZTO NWs have the best degradation efficiency. Figure 5d reveals the change of MB concentration versus time. The red line denotes ZTO NWs, which degraded 85% of MB in 120 min; the blue line denotes 1 at% Ag-ZTO NWs, which degraded 94% of MB in 120 min and the green line denotes 3 at% Ag-ZTO NWs, degrading 80% of MB in the first 60 min and degrading 96% in 120 min. To obtain a better understanding of the kinetics of photodegradation, pseudo-first-order equation was used as shown in Equation (6) [40]:

(6) $$\ln(\frac{C_{0}}{C_{t}})=\kappa t$$

where κ is the rate constant and t means time. Figure 5e shows the comparison of the rate constants for ZTO NWs, 1 at% Ag-ZTO NWs and 3 at% Ag-ZTO NWs, which were 0.0157, 0.023 and 0.025 min^−1^, respectively. These results demonstrate the enhanced photodegradation performance with the silver modification. 

#### 3.4.2. Effect of Reactive Free Radicals

To understand the influence of different reactive free radicals in the photodegradation process, the reaction of each free radical was inhibited by adding scavengers [41,42]. The 3 at% Ag-ZTO NWs were added to ethylenediaminetetraacetic acid (EDTA), tert-butanol (TBA) and benzoquinone (1,4-benzoquinone, BQ) as the scavengers of h^+^, ·OH and ·O_2_^−^, respectively. EDTA was used as a scavenger of h^+^. The reaction between h^+^ and EDTA is as follows: EDTA + h^+^ → CO_2_ + H_2_O. TBA was used as a scavenger of ·OH. BQ was used as a scavenger of ·O_2_^−^, which can trap superoxide anions via an electron transfer mechanism: BQ +·O_2_^−^ → ·BQ^−^ + O_2_. Figure 5f shows the change in the photodegradation efficiency after adding different scavengers. The original efficiency of 3 at% Ag-ZTO NWs was 96% in 120 min; after adding EDTA, TBA and BQ, the efficiency was reduced to 15%, 52% and 63%, respectively. The results indicate that adding EDTA led to the highest inhibition, followed by TBA and BQ. The degree of effects for reactive species in photodegradation is h^+^ >·OH >·O_2_^−^ with the major mechanisms shown in Equations (7)–(9):

Zn_2_SnO_4_ + hv (≥E_g_) → Zn_2_SnO_4_ (h_cb_^+^) + Zn_2_SnO_4_ (e^−^) (7)

Zn_2_SnO_4_ (h_cb_^+^) + OH^−^ → ·OH (8)

(h_cb_^+^, ·OH) + Dye (MB) → CO_2_ +H_2_O + degraded products (9)

#### 3.4.3. Reliability and Stability

To study the stability of ZTO NWs and Ag-ZTO NWs, three consecutive cycles of MB degradation were conducted. Figure S9c shows that the photodegradation efficiencies were 96%, 84% and 82%, respectively. The decrease in degradation rate may be attributed to the photo-corrosion of the nanowire surface or the accumulation of contaminants on the nanowire surface; however, the degradation efficiency of the second and third times tends to be stable. As previously reported [12,13,14], the degradation efficiency of MB decreases by 10–20% before the photocatalyst is reused, while our degradation efficiency dropped about 14% and became stable following the typical curve, indicating that 3 at% Ag-ZTO NWs are expected to be a reusable photocatalyst. Additionally, the XRD pattern in Figure S9d reveals that the ZTO NWs still had great crystallinity after multiple cycles.

#### 3.4.4. Photodegradation Mechanism

Figure 5h is the schematic illustration for the photodegradation mechanism of Ag-ZTO NWs. As the ZTO NWs were illuminated, the electrons in the valence band were excited to the conduction band to form electron-hole pairs at the NW surface. Electrons in the conduction band reacted with oxygen to form superoxide radicals (∙O_2_^−^), while the holes in the valence band could react with pollutants and the hydroxide ions (OH^-^) to generate hydroxyl radicals (∙OH), thereby degrading the pollutants. With the ZTO NWs modified with silver, the excited electrons diffused to the silver particles [43], reducing the recombination rate of electron-hole pairs. The PL intensity is proportional to the electron-hole recombination rate; thus, the PL intensity decreased after Ag modification. When the recombination efficiency of electron-hole pairs was reduced, the electrons and holes had more opportunities to react with pollutants, increasing the degradation rate.

## 4. Conclusions

Single crystalline Zn_2_SnO_4_ (ZTO) nanowires were successfully synthesized by chemical vapor deposition and selective etching. Silver nanoparticles were modified on the surface of ZTO nanowires using the chemical bath redox method to fabricate 1 at% Ag-ZTO NWs and 3 at% Ag-ZTO NWs. The structures of ZTO NWs and Ag-ZTO NWs were confirmed by EDS, XRD, XPS and HRTEM. The PL intensity decrease after surface modification reflects the decrease in the electron-hole pair recombination efficiency, which is beneficial for photocatalysis. The single ZTO NW shows low electrical resistivity of 6.01 × 10^−5^ Ω·m. A total of 96% of MB was degraded by 3 at% Ag-ZTO NWs in 120 min. h^+^ and ·OH were the main reactants of photodegradation. After multiple cycles, the nanowires maintained degradation efficiency well without apparent change in terms of structure and morphology, showing reliability in photocatalysis.

## Figures and Tables

**Table 1:** Electrical resistivity measurements of ZTO NW, 1 at% Ag-ZTO NW and 3 at% Ag-ZTO NW.

	ZTO NW	1 at% Ag-ZTO NW	3 at% Ag-ZTO NW
**Resistivity**	6.01 × 10^−5^ Ω·m	2.1 × 10^−4^ Ω·m	4.3 × 10^−4^ Ω·m

## Data Availability

Not applicable.

## Supplementary Materials

The following supporting information can be downloaded at: https://www.mdpi.com/article/10.3390/nano12071201/s1, Figure S1: Schematic illustration for fabrication of Ag-ZTO NWs. Figure S2: SEM images and XRD pattern of ZTO NWs before and after selective etching. (a) SEM image before selective etching (b) SEM image after selective etching (c) comparison of XRD patterns before and after selective etching. Figure S3: Schematic illustration of growth mechanism for ZTO NWs. Figure S4: EDS and mapping images of 1 at% Ag-ZTO NW and 3 at% Ag-ZTO NW. (a) EDS analysis of 1 at% Ag-ZTO NW (b) EDS analysis of 3 at% Ag-ZTO NW (c) mapping images of 1 at% Ag-ZTO NW (d) mapping images of 3 at% Ag-ZTO NW. Figure S5: Electrical measurement setup for ZTO NWs and Ag-ZTO NWs. (a) Schematic illustration of the electrical measurements. SEM images of (b) ZTO NW (c) 1 at% Ag-ZTO NW (d) 3 at% Ag-ZTO NW connected to 4 electrodes. Figure S6: I–V measurements of single ZTO nanowire (a) R_13+_ (b) R_13−_ (c) R_14+_ (d) R_14−_ (e) R_23+_ (f) R_23−_ (g) R_24+_ (h) R_24−_. Figure S7: I–V measurements of single 1 at% Ag-ZTO nanowire (a) R_13+_ (b) R_13−_ (c) R_14+_ (d) R_14−_ (e) R_23+_ (f) R_23−_ (g) R_24+_ (h) R_24−_. Figure S8: I–V measurements of single 3 at% Ag-ZTO nanowire (a) R_13+_ (b) R_13−_ (c) R_14+_ (d) R_14−_ (e) R_23+_ (f) R_23−_ (g) R_24+_ (h) R_24−_. Figure S9: Photocatalytic reliability of ZTO NWs and Ag-ZTO NWs. (a) Degradation behaviors of ZTO NWs for three repeated cycles (b) degradation behaviors of 1 at% Ag-ZTO NWs for three repeated cycles (c) degradation behaviors of 3 at% Ag-ZTO NWs for three repeated cycles (d) XRD pattern after the third cycle of degradation.
