# Supplementary material for: Fabrication and Photocatalytic Properties of Zinc Tin Oxide Nanowires Decorated with Silver Nanoparticles
Source: Nanomaterials (Basel). 2022 Apr 3;12(7):1201. doi: 10.3390/nano12071201 (PMC9000500; doi:10.3390/nano12071201)
Supplement: Supplementary file 1 [file nanomaterials-12-01201-s001.zip › nanomaterials-1642819-supplementary.pdf]

Supporting Information

Fabrication and Photocatalytic Properties of Zinc Tin Oxide Nanowires Decorated with Silver Nanoparticles

Jia-Chi Su[1], Tsung-Lin Hsieh[1], Shu-Meng Yang[1], Shao-Chun Chao[1] and Kuo-Chang Lu[1,2]*

1 Department of Materials Science and Engineering, National Cheng Kung University, Tainan 701, Taiwan; n56081412@ncku.edu.tw (J.-C.S.); n56091255@gs.ncku.edu.tw (T.-L.H.); n56074287@gs.ncku.edu.tw (S.-M.Y.); n56104375@gs.ncku.edu.tw (S.-C.C.)

2 Core Facility Center, National Cheng Kung University, Tainan 701, Taiwan

* Correspondence: gkclu@mail.ncku.edu.tw; Tel.: +886-6-275-7575 (ext. 62920)

List of contents

Figure S1｜Schematic illustration for fabrication of Ag-ZTO NWs

Figure S2｜SEM images and XRD pattern of ZTO NWs before and after selective etching. (a)SEM image before selective etching (b)SEM image after selective etching (c)comparison of XRD patterns before and after selective etching.

Figure S3｜Schematic illustration of growth mechanism for ZTO NWs

Figure S4｜EDS and mapping images of 1 at% Ag-ZTO NW and 3 at% Ag-ZTO NW. (a)EDS analysis of 1 at% Ag-ZTO NW (b) EDS analysis of 3 at% Ag-ZTO NW (c) mapping images of 1 at% Ag-ZTO NW (d) mapping images of 3 at% Ag-ZTO NW.

Figure S5｜Electrical measurements setup for ZTO NWs and Ag-ZTO NWs. (a)Schematic illustration of the electrical measurements. SEM images of (b)ZTO NW (c)1 at% Ag-ZTO NW (d)3 at% Ag-ZTO NW connected to 4 electrodes.

Figure S6｜I-V measurements of single ZTO nanowire (a)R_{13+} (b)R_{13-} (c)R_{14+} (d)R_{14-}(e)R_{23+} (f)R_{23-} (g)R_{24+} (h)R_{24-}.

Figure S7｜I-V measurements of single 1 at% Ag-ZTO nanowire (a)R_{13+} (b)R_{13-} (c)R_{14+} (d)R_{14-}(e)R_{23+} (f)R_{23-} (g)R_{24+} (h)R_{24-}.

Figure S8｜I-V measurements of single 3 at% Ag-ZTO nanowire (a)R_{13+} (b)R_{13-} (c)R_{14+} (d)R_{14-}(e)R_{23+} (f)R_{23-} (g)R_{24+} (h)R_{24-}.

Figure S9｜Photocatalytic reliability of ZTO NWs and Ag-ZTO NWs. (a)degradation behaviors of ZTO NWs for three repeated cycles (b)degradation behaviors of 1 at% Ag-ZTO NWs for three repeated cycles (c)degradation behaviors of 3 at% Ag-ZTO NWs for three repeated cycles (d)XRD pattern after the 3rd cycle of degradation.

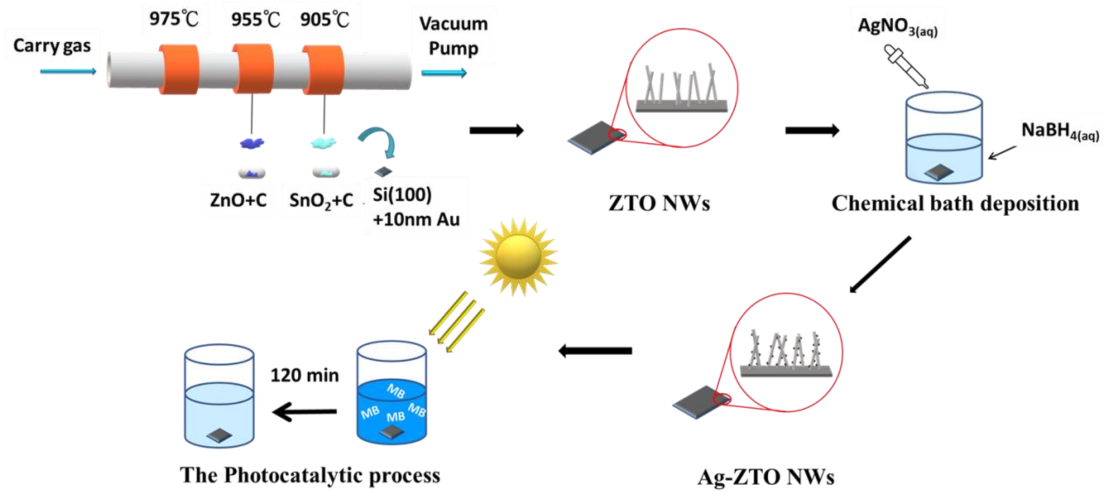

Figure S1. Schematic illustration for fabrication of Ag-ZTO NWs

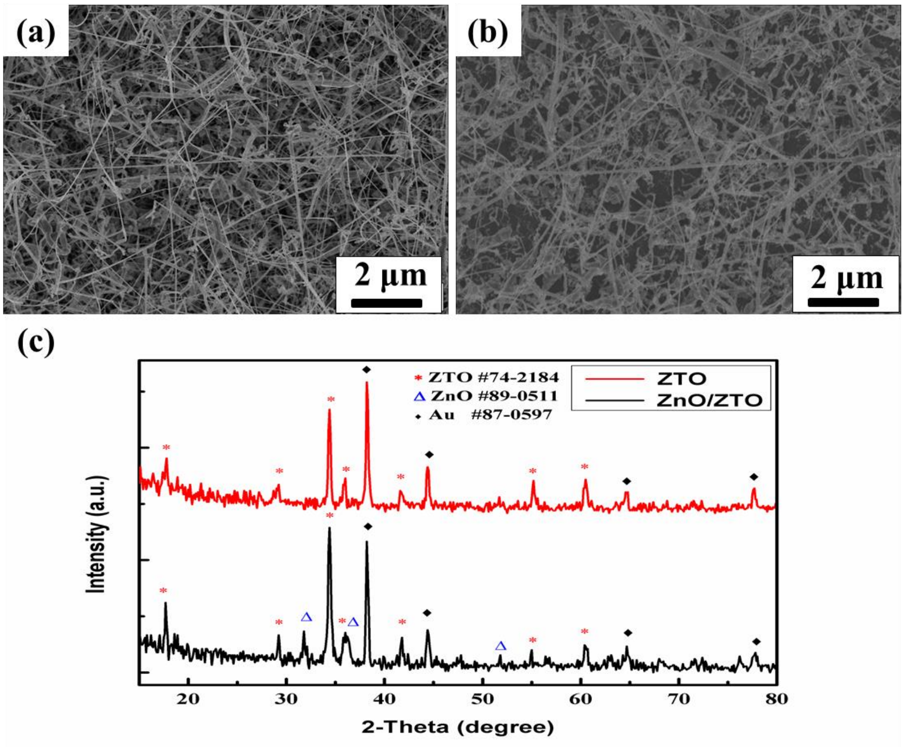

Figure S2. SEM images and XRD pattern of ZTO NWs before and after selective etching. (**a**)SEM image before selective etching (**b**)SEM image after selective etching (**c**)comparison of XRD patterns before and after selective etching.

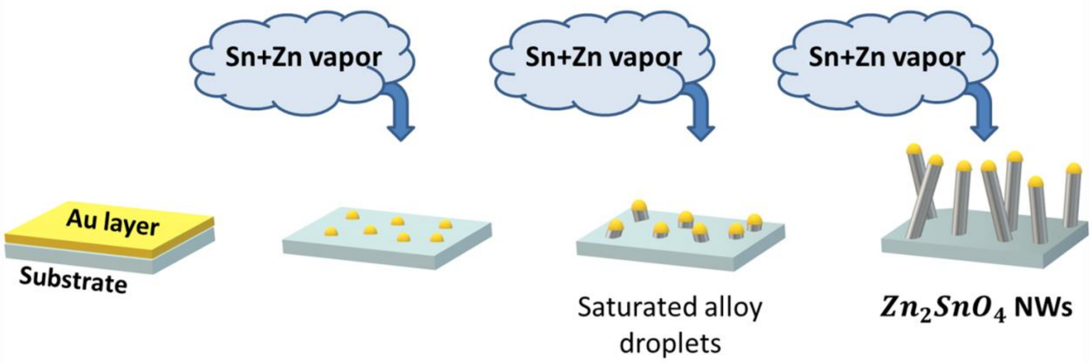

Figure S3. Schematic illustration of growth mechanism for ZTO NWs

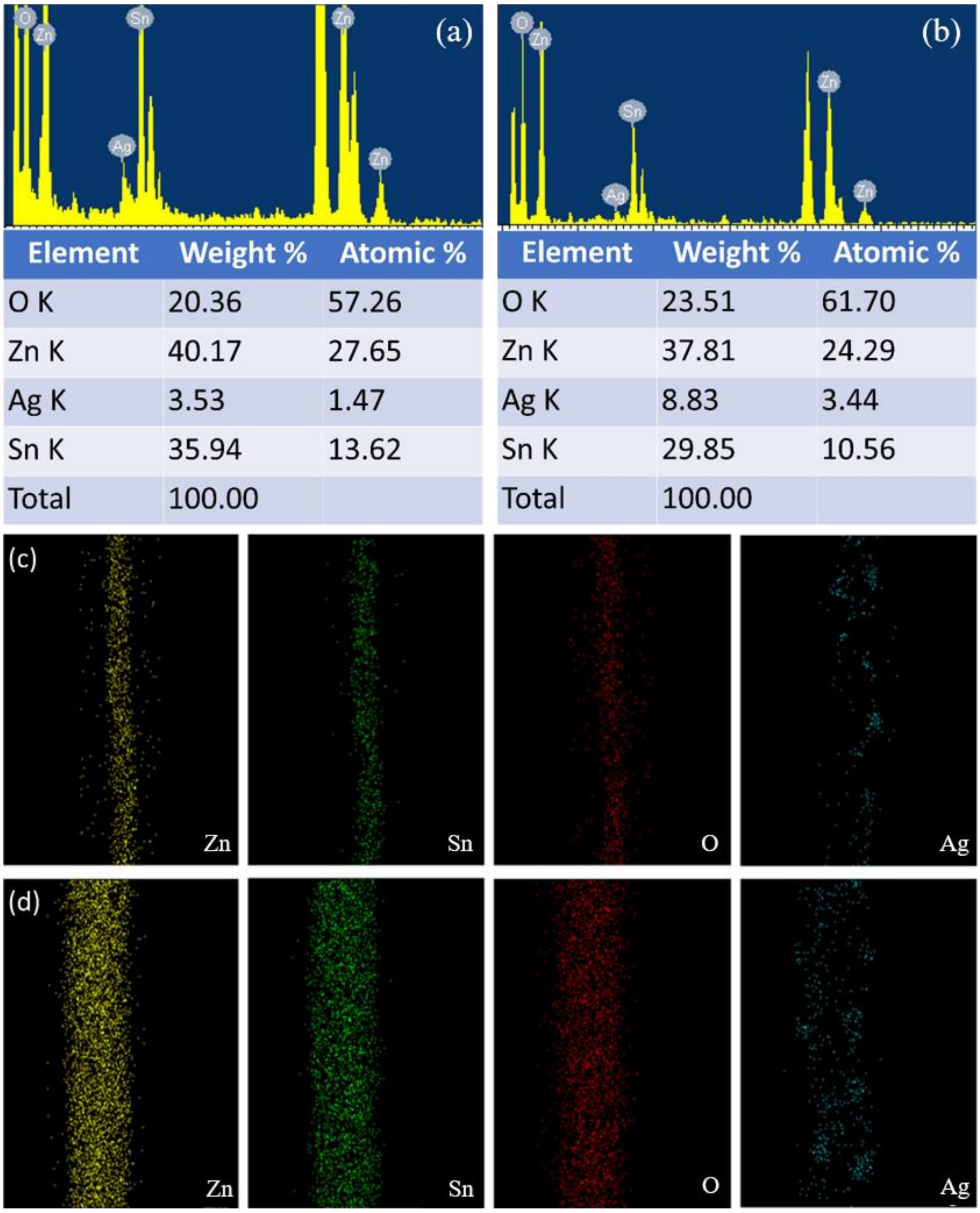

Element	Weight %	Atomic %
O K	20.36	57.26
Zn K	40.17	27.65
Ag K	3.53	1.47
Sn K	35.94	13.62
Total	100.00	

Element	Weight %	Atomic %
O K	23.51	61.70
Zn K	37.81	24.29
Ag K	8.83	3.44
Sn K	29.85	10.56
Total	100.00	

Figure S4. EDS and mapping images of 1 at% Ag-ZTO NW and 3 at% Ag-ZTO NW. (**a**)EDS analysis of 1 at% Ag-ZTO NW (**b**) EDS analysis of 3 at% Ag-ZTO NW (**c**) mapping images of 1 at% Ag-ZTO NW (**d**) mapping images of 3 at% Ag-ZTO NW.

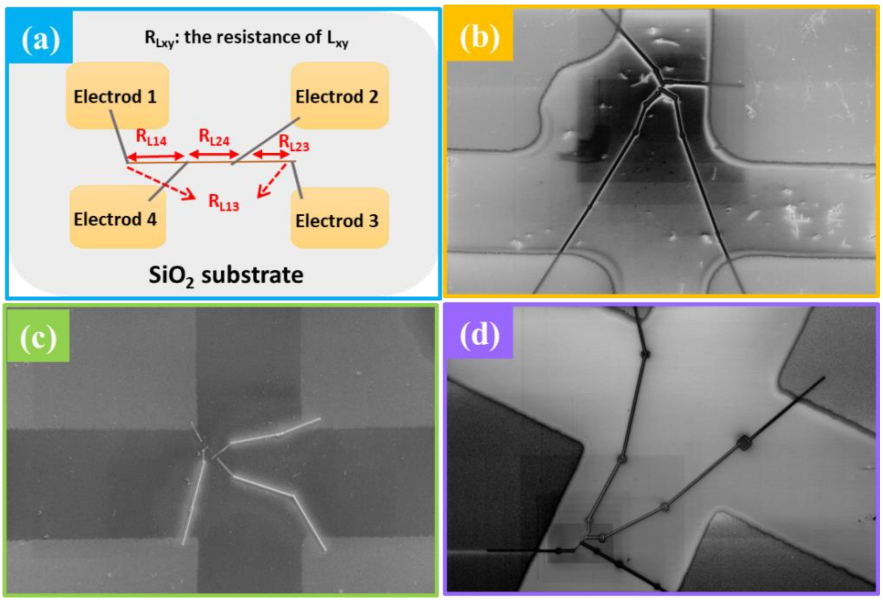

Figure S5. Electrical measurements setup for ZTO NWs and Ag-ZTO NWs. (**a**)Schematic illustration of the electrical measurements. SEM images of (**b**)ZTO NW (**c**)1 at% Ag-ZTO NW (**d**)3 at% Ag-ZTO NW connected to 4 electrodes.

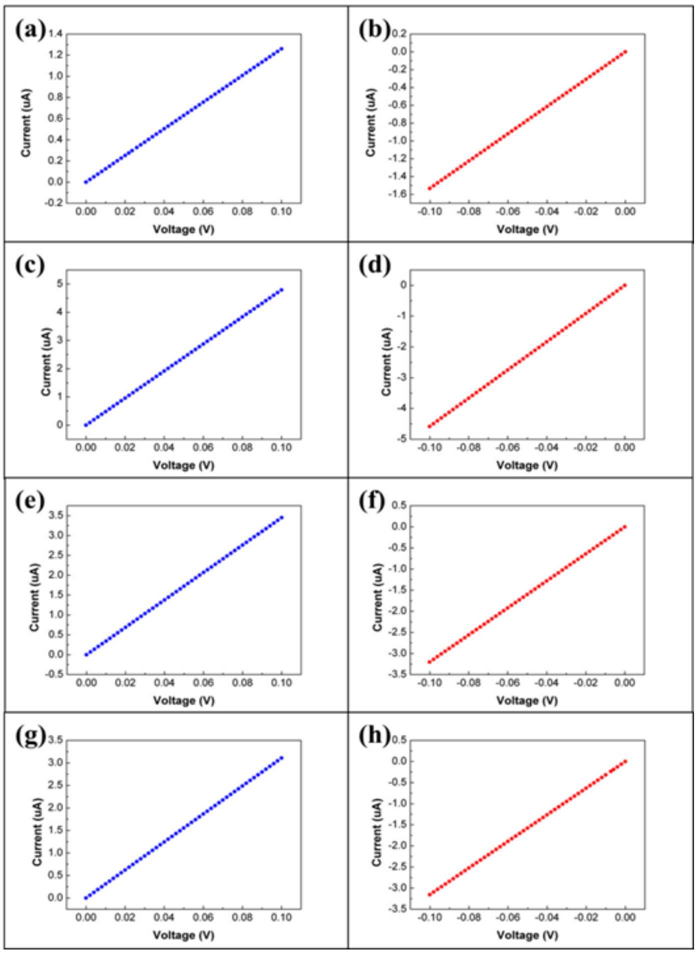

Figure S6. I-V measurements of single ZTO nanowire (**a**)R_{13+} (**b**)R_{13-} (**c**)R_{14+} (**d**)R_{14-}(**e**)R_{23+} (**f**)R_{23-} (**g**)R_{24+} (**h**)R_{24-}.

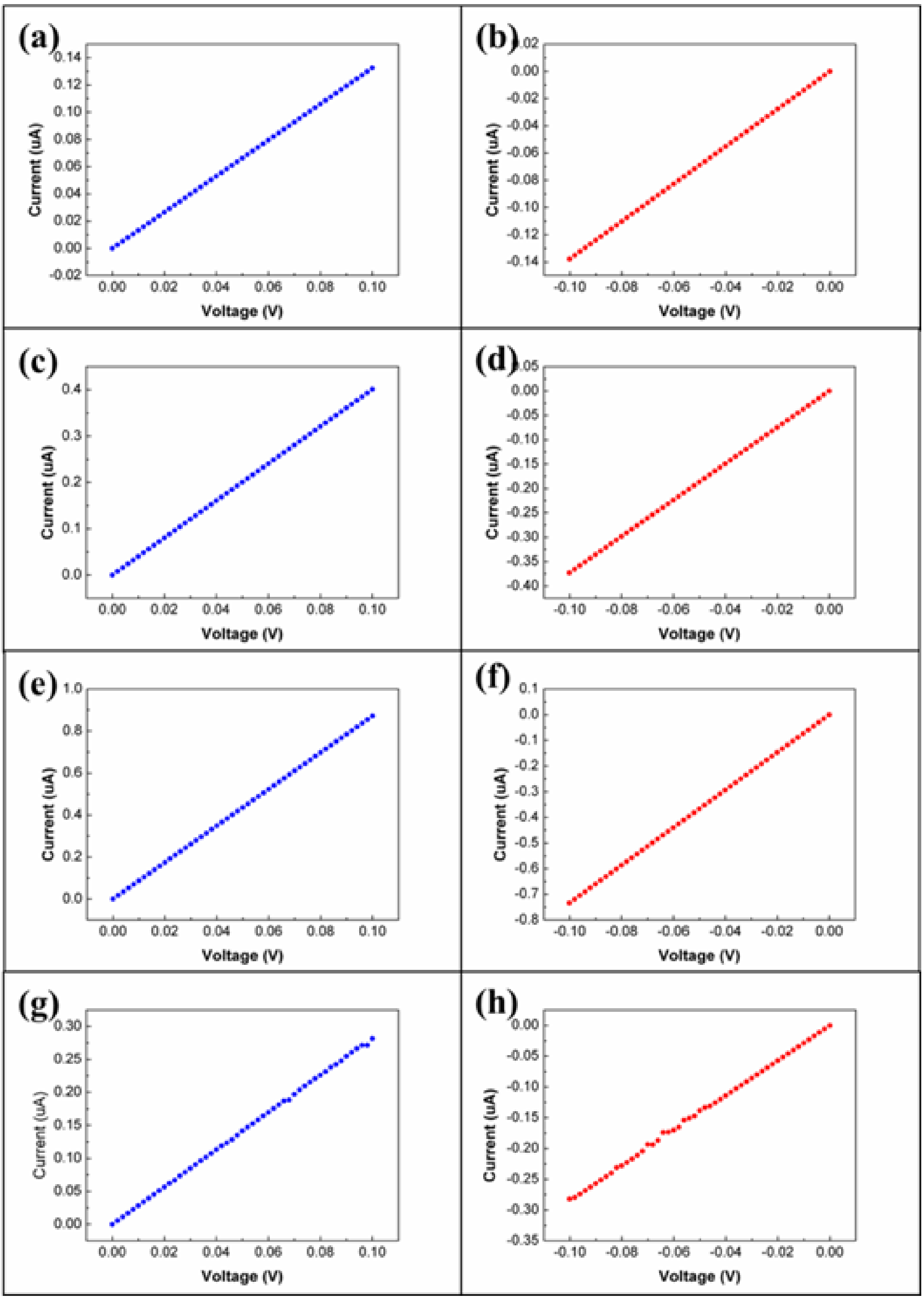

Figure S7. I-V measurements of single 1 at% Ag-ZTO nanowire (**a**)R_{13+} (**b**)R_{13-} (**c**)R_{14+} (**d**)R_{14-}(**e**)R_{23+} (**f**)R_{23-} (**g**)R_{24+} (**h**)R_{24-}.

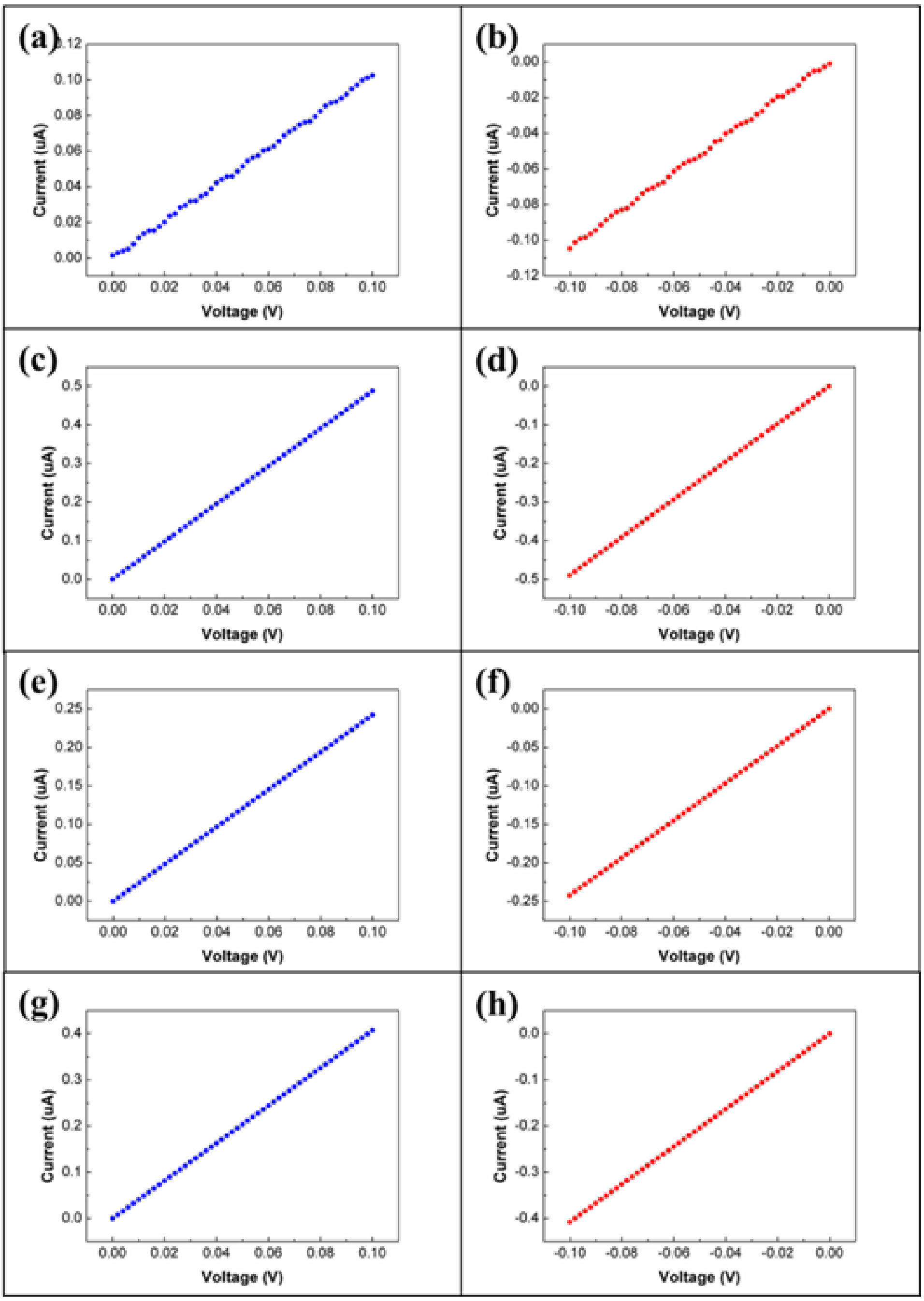

Figure S8. I-V measurements of single 3 at% Ag-ZTO nanowire (**a**)R_{13+} (**b**)R_{13-} (**c**)R_{14+} (**d**)R_{14-}(**e**)R_{23+} (**f**)R_{23-} (**g**)R_{24+} (**h**)R_{24-}.

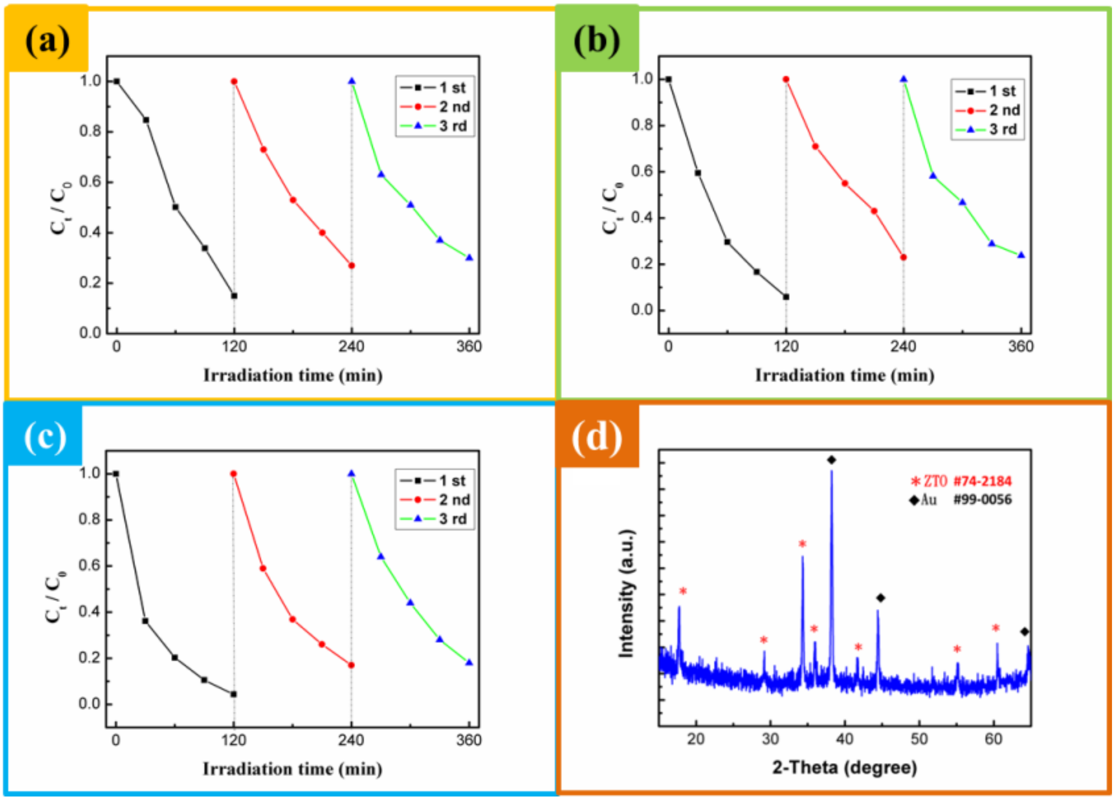

Figure S9. Photocatalytic reliability of ZTO NWs and Ag-ZTO NWs. (**a**)degradation behaviors of ZTO NWs for three repeated cycles (**b**)degradation behaviors of 1 at% Ag-ZTO NWs for three repeated cycles (**c**)degradation behaviors of 3 at% Ag-ZTO NWs for three repeated cycles (**d**)XRD pattern after the 3rd cycle of degradation.
